# Fatal drowning in Indonesia: understanding knowledge gaps through a scoping review

**DOI:** 10.1093/heapro/daad130

**Published:** 2023-10-18

**Authors:** Muthia Cenderadewi, Susan G Devine, Dian Puspita Sari, Richard C Franklin

**Affiliations:** College of Public Health, Medical and Veterinary Sciences, James Cook University, Bebegu Yumba Campus, Douglas, QLD 4811, Australia; Medical Faculty, University of Mataram, Mataram, West Nusa Tenggara 83126, Indonesia; College of Public Health, Medical and Veterinary Sciences, James Cook University, Bebegu Yumba Campus, Douglas, QLD 4811, Australia; Medical Faculty, University of Mataram, Mataram, West Nusa Tenggara 83126, Indonesia; College of Public Health, Medical and Veterinary Sciences, James Cook University, Bebegu Yumba Campus, Douglas, QLD 4811, Australia; Royal Life Saving Society – Australia, Broadway, NSW 2007, Australia

**Keywords:** Drowning, prevention, risk factors, epidemiology, injury, health promotion, health policy, health promoting policies, evidence-based health promotion, low- and middle-income countries, Indonesia

## Abstract

Little is known about unintentional drowning deaths in Indonesia, the world’s fourth most populous and largest archipelagic country. This study aimed to describe the epidemiology and risk factors of unintentional drowning in Indonesia and explore existing health promotion and drowning prevention approaches in Indonesia within a socio-ecological health promotion framework. A scoping review, guided by PRISMA-ScR, was conducted to locate peer-reviewed studies and government reports/policy documents published until May 2023, in English or Indonesian language, using MEDLINE (Ovid), CINAHL, Informit, PsycINFO (ProQuest), Scopus, SafetyLit, BioMed Central and Google Scholar, Indonesian journal databases (Sinta, Garuda) and government agencies websites around the terms: drown, swim, flood, hurricane, cyclone, disaster, water rescue and maritime/boat safety. This review identified 32 papers. However, a paucity of information on unintentional drowning rates, risk factors and prevention in Indonesia was noted. The unavailability of a coordinated national drowning data collection system in Indonesia, from which national and subnational subcategory data can be collected, underlines the possibility of under-representation of drowning mortality. The association between various exposures and drowning incidents has not been fully investigated. An over-reliance on individual-focused, behaviour-based, preventive measures was observed. These findings highlight the need for improving drowning surveillance to ensure the availability and reliability of drowning data; and strengthening research to understand the risk factors for drowning and delivery of drowning prevention programs. Further policy development and research focusing on health promotion approaches that reflect a socio-ecological approach to drowning prevention in Indonesia is imperative.

**Contribution to Health Promotion**

- By upholding concepts of health promotion, this review may inform the international health promotion community on potential challenges of drowning prevention efforts in resource-limited settings.
- The over-reliance on educational interventions aimed at individuals and the limited availability of population-focused drowning prevention measures is observed in Indonesia.
- The research area of community participation in creating safe environment and evidence-informed water safety and drowning prevention-related policy development is relatively neglected in Indonesia.
- A demand for further research focused on policy formulation, implementation and evaluation to prevent drowning across low- and middle-income countries is apparent.

## BACKGROUND

Drowning is the third leading cause of death by unintentional injury worldwide, after road injury and falls (Ahlm *et al.*, 2013; World Health Organization, 2014, 2016; Mokdad *et al.*, 2016; Franklin *et al.*, 2020). Most drowning deaths worldwide (91%, or 337,240 drowning deaths) occurred in low- and middle-income countries (LMICs), particularly in Southeast Asia (35%, or 130,149 drowning deaths), underlining the importance of providing reliable information on unintentional drowning deaths to inform the development, implementation and evaluation of drowning prevention interventions and policy within countries in this region (World Health Organization, 2014). However, understanding of unintentional drowning deaths in Indonesia, the world’s largest archipelagic and fourth most populous country with high numbers of meteorological and hydrological events, is limited (Cenderadewi *et al.*, 2020). To address the global burden of drowning, effective health promotion approaches which address drowning at all levels will be required, and comprehensive information on drowning incidents, including in Indonesia, is vital for the planning and implementation of relevant prevention approaches.

Indonesia is one of the world’s most natural disaster-prone areas and is at risk from multiple hazards, including floodings, landslides, earthquakes, tsunamis, volcanic activities and tropical cyclones (Global Facility for Disaster Reduction and Recovery, 2019). Indonesia is also the largest archipelagic state worldwide, consisting of 16 056 islands, extending 5150 kilometres between the Indian and Pacific Oceans (Global Facility for Disaster Reduction and Recovery, 2019; Indonesian National Bureau of Statistics, 2019; United Nations, 2019; Embassy of Indonesia for the United States of America, 2019). Understanding the potential contribution of water transport-related and disaster-related drowning deaths in Indonesia on top of the rate of unintentional drowning will be important, as they are often left out of studies on drowning worldwide (Sindall *et al.*, 2022).

The prevention of drowning encompasses a wide range of measures, ranging from individual-focused approaches, such as swimming training programmes, to community-based actions, such as community participation in controlling access to open water bodies, policy development on water safety regulations and providing equitable access to safe drinking water (Crawford *et al.*, 2014; Leavy *et al.*, 2015, 2016, 2023). This emphasizes the urgent need for cohesive approaches across the spectrum of upstream, midstream, and downstream drowning prevention (Linnan *et al.*, 2012, 2013; World Health Organization, 2014; Guevarra *et al.*, 2015; Leavy *et al.*, 2023).

Drowning prevention is closely linked to health and safety promotion (Plitponkarnpim and Chinapa, 2014). By connecting drowning prevention and health promotion, a broader understanding of drowning prevention can be achieved, which includes the process of empowering individuals and communities in taking control over their own health-related behaviours and practices (Leavy *et al.*, 2016, 2023; Giles *et al.*, 2017; World Health Organization Regional Office for the Eastern Meditteranean, 2017). Talbot and Verrinder (2017) illustrated the concepts of health promotion in the Health Promotion Framework, which comprises of (i) medical approaches, focusing on individual risk assessment and health information; (ii) behavioural approaches, focusing on health education, skills development and social marketing; and (iii) socio-environmental approaches, focusing on increasing cross-sector partnerships and community capacity, including community action, community participation, structural change, policy development and review and economic and regulatory activities. This framework will be used throughout this review to assess gaps in drowning prevention approaches in Indonesia (Talbot and Verrinder, 2017).

### Research aims

This review aimed to describe the epidemiology and risk factors of unintentional drowning in Indonesia and explore health promotion and prevention approaches currently in place.

### Research questions

This study answered the following questions:

1. What information is available on fatal unintentional drowning mortality numbers and rates in Indonesia?
2. What is known about risk factors for unintentional drowning in Indonesia?
3. What prevention and health promotion approaches are currently being used in Indonesia to reduce unintentional drowning deaths?
4. How can the Health Promotion Framework (Talbot and Verrinder, 2017) be applied to inform strategy development to prevent unintentional drowning Indonesia?

## METHODS

This review was conducted using a scoping review methodology guided by the Arksey and O’Malley methodological framework, Joanna Briggs Institute (JBI) guideline, and the Preferred Reporting Items for Systematic Reviews and Meta-Analyses extension for Scoping Reviews (PRISMA-ScR) (Arksey and O’Malley, 2005; Tricco *et al.*, 2018; Joanna Briggs Institute, 2020; McGowan *et al.*, 2020; Rethlefsen *et al.*, 2021). Scoping review methodology was selected to explore characteristics of unintentional drowning in Indonesia, a little studied area of interest, and to map evidence from both research and non-research sources, providing a comprehensive overview on unintentional drowning in Indonesia and knowledge gaps for subsequent evidence syntheses (Joanna Briggs Institute, 2020).

### Search strategy

A systematic search was conducted in May 2023 to identify relevant literature for all categories of unintentional drowning published to this date. Eight databases were searched including MEDLINE (Ovid), CINAHL, Informit, PsycINFO (ProQuest), Scopus, SafetyLit, BioMed Central and Google Scholar; along with two Indonesian journal databases (Sinta and Garuda); and websites of Indonesian government agencies identified in previous studies as the most important actors for documenting drowning events and undertaking drowning prevention in Indonesia, including for disaster- and water transport-related drowning: (i) Ministry of Health; (ii) Ministry of Education, Culture, Research, and Technology; (iii) Ministry of Marine Affairs and Fisheries; (iv) Ministry of Transportation; (v) National Disaster Management Agency and (vi) National Search and Rescue Agency (Andry and Yuliani, 2014; Hapsari and Zenurianto, 2016; Buchori *et al.*, 2018; Handayani *et al.*, 2019; Isa *et al.*, 2019; Indonesian Ministry of Health, 2020; Mantong *et al.*, 2020). The searches were undertaken in English and Indonesian language, using the most relevant and exhaustive search terms, in accordance with each database utilized (for details on search terms, see Supplementary Appendix 1).

Inclusion and exclusion criteria were used to narrow down the systematic search corresponding to the research objectives. Inclusion criteria included (i) literature published up until May 2023; (ii) original research articles; (iii) comprehensive scientific reviews, meta-analyses, statements of clinical standards, case reports, opinion pieces; (iv) grey literature, such as government or other authoritative reports, policy statements, issues papers, theses and dissertations and conference papers/proceedings; (v) full-text available; (vi) published in English or Indonesian language; (vii) drowning deaths in humans; (viii) drowning specifically taking place in Indonesia; (ix) unintentional drowning deaths, including accidental, disaster-related and water transport-related drowning deaths; (x) drowning risk factors; (xi) drowning prevention; (xii) regulations on water safety, safe boating and shipping, maritime safety and disaster risk management relevant to drowning prevention and (xiii) health promotion approaches on water safety and drowning prevention. The exclusion criteria excluded all publications on tsunami-related events, intentional drowning (intentional self-harm by drowning, assault by drowning, drowning by undetermined intent), and drowning in war operations.

Unintentional drowning deaths screened in this review included accidental drowning deaths, disaster-related drowning deaths and water transport-related drowning deaths. This inclusion was based on the ICD 10 coding for non-intentional drowning, which includes accidental drowning (W65-W74), disaster-related drowning (X34-X39), and water transport-related drowning (V90.-, V92.-); while excluding intentional self-harm by drowning (X71), assault by drowning (X92), drowning by undetermined intent (Y21) and drowned in war operations NOS (Y36.4) (World Health Organization, 2019). Studies on the epidemiology of unintentional drowning were included in this review if they reported the epidemiological measures of drowning mortality as a count, proportion or rate. When calculable, mortality rates were inferred from drowning death counts reported in the reviewed studies. Drowning deaths involving a foreign national that occurred in Indonesia were also included. Conversely, instances where Indonesian citizens drowned abroad were excluded.

Despite being disaster-related, tsunami-related drowning deaths were excluded, due to its relatively rare occurrence (only 75 tsunami events, out of 41 470 disaster events, were documented in Indonesia between 1815 and 2023 (Indonesian National Disaster Management Agency., 2023a)), and the low chance of victim survival, even for good swimmers, due to the sheer force of tsunami vortices (Kurisu *et al.*, 2018), underlining the fact that risk factors and prevention of tsunami-related drowning are entirely distinct from those of other causes (Doocy *et al.*, 2007, 2009). Moreover, even though our database searches included papers on boating, shipping and maritime safety and disaster risk management in Indonesia, all studies identified were on regulatory activities and community participation around maritime safety and flood mitigation not specifically linked nor relevant to the efforts of preventing drowning, hence they were excluded. However, it is acknowledged that disaster risk management, including tsunami risk assessment and preparedness, boating, shipping and maritime safety and their link to drowning prevention, are an area of concern urgently needed to be further investigated in Indonesia (Muis *et al.*, 2015; Djalante and Garschagen, 2017; Sari and Prayoga, 2018; Mantong *et al.*, 2020).

### Review strategy

Publications were screened for inclusion by title, abstract and full text. Two researchers (M.C. and D.P.S.) independently reviewed search results at the stage of title, abstract and full-text review. The flow of the selection process of all identified records was as follows:

#### Title and abstract screening

The titles and abstracts of all records identified through the database searching were screened by the inclusion and exclusion criteria, to ensure the relevance of the studies included for the evidence-informed review.

#### Full-text screening

**Fig. 1:**
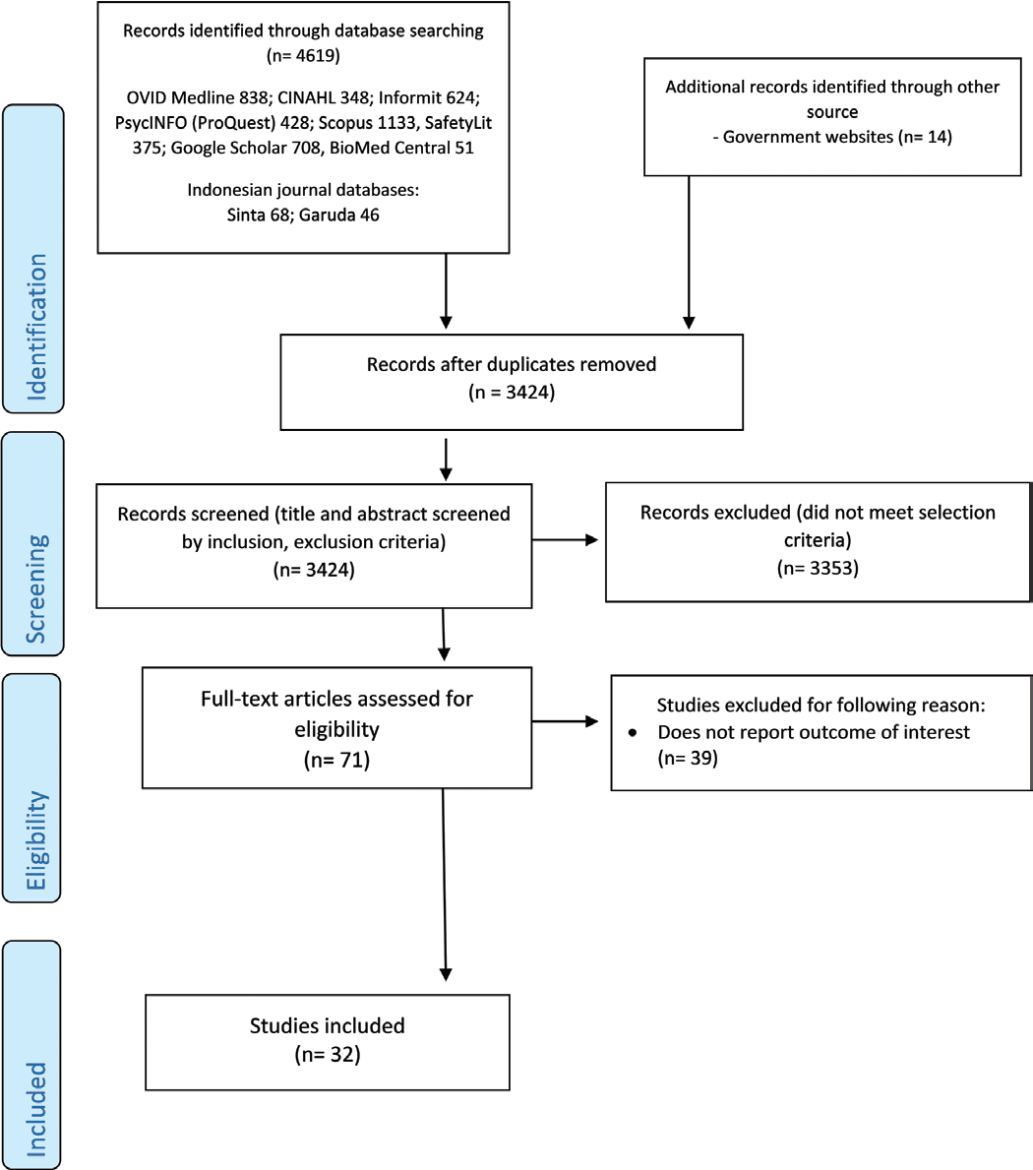
The PRISMA-ScR 2020 flow diagram for scoping review.

Full-text versions of identified articles were then appraised to examine the relevance of the finding of the studies to answer the research questions. The PRISMA-ScR flow diagram summarized the review process, as presented in **Figure 1**.

### Data abstraction

The following data were abstracted from the original peer-reviewed publications: authors, year of study and publication, type of publication (original research article, comprehensive scientific review, meta-analysis, statement of clinical standards, case report, opinion piece or grey literature), data source, study aim, study design, study sample and setting, scale of study (national or subnational), categories of drowning investigated, methods, relevant findings on the mortality rate, risk factors, and prevention of drowning, intervention type and comparator (if applicable). For grey literature, the following data were abstracted: authors (government agency), year of publication, scale of study, categories of drowning investigated and relevant findings. Details of data abstracted from reviewed studies are shown in the online Supplementary Appendix 2.

### Synthesis/analysis

Mortality numbers, proportions and rates were extracted or inferred from the identified studies as epidemiological measures of unintentional drowning, either at the subnational or national scale. For this review, the Health Promotion Framework (Talbot and Verrinder, 2017), which comprises medical, behavioural and socio-environmental approaches at the individual through to the population level, was used to map the socio-ecological dimension of drowning prevention and health promotion approaches that have been or are currently being used in Indonesia.

### Ethics approval

Ethical approval has been obtained from the Human Research Ethics Committee of the University of Mataram—Indonesia (Ethics Approval number 128/UN18.F8/ETIK/2023). The scoping review did not collect personal, sensitive or confidential information from participants, and only used publicly accessible documents as evidence.

## RESULTS

From the 4619 potentially relevant records initially identified via database searching and government websites, 32 articles were included in this review, including 24 (75%) peer-reviewed publications (Suwardjo *et al.*, 2010; Usaputro and Yulianti, 2013; Wulur, 2013; Gobel *et al.*, 2014; Astreani and Alit, 2015; Prasetyo, 2017; Hu *et al.*, 2018; Lesmana *et al.*, 2018; Sillehu and Kartika, 2018; Patimah, 2019; Patimah *et al.*, 2019; Elsi and Gusti, 2020; Hady *et al.*, 2020; Nugroho and Suryono, 2020; Ose *et al.*, 2020; Rosmi *et al.*, 2020; Suryono and Nugroho, 2020; Faradisi *et al.*, 2021; Saputra, 2021; Sugiantoro and Wahyudi, 2021; Sukarna *et al.*, 2021; Welembuntu *et al.*, 2021; Fernalia *et al.*, 2022; Pranoto *et al.*, 2023; Sadewa *et al.*, 2023) and eight (25%) pieces of grey literature consisting of government issues papers, theses and a conference proceeding (Indonesian Ministry of Health, 2015, 2017, 2020; Prasetyo, 2017; Widyastuti and Rustini, 2017; Nadapdap, 2021; Indonesian National Disaster Management Agency, 2023a, 2023b). All but one reviewed articles were published in the Indonesian language. All but one peer-reviewed studies were published in local and national Indonesian journals in Indonesian language. Details of reviewed studies are presented in the Supplementary Appendix 2.

Of the 32 papers reviewed in this study, the information on the number of unintentional drowning deaths in Indonesia at the national and provincial level was only presented in eight (25%) studies, including three (9.4%) studies reporting accidental drowning deaths (Usaputro and Yulianti, 2013; Wulur, 2013; Astreani and Alit, 2015), two (6.3%) papers reporting disaster-related deaths (Hu *et al.*, 2018; Indonesian National Disaster Management Agency, 2023), and three (9.4%) papers reporting water transport-related deaths (Suwardjo *et al.*, 2010; Saputra, 2021; Indonesian National Disaster Management Agency, 2023). Risk factors were investigated in 11 (34.4%) studies, with seven (21.9%) studies discussing factors potentially contributing to accidental drowning events (Usaputro and Yulianti, 2013; Wulur, 2013; Astreani and Alit, 2015; Prasetyo, 2017; Widyastuti and Rustini, 2017; Elsi and Gusti, 2020; Welembuntu *et al.*, 2021), three (9.4%) on contributing factors to water transport-related deaths (Suwardjo *et al.*, 2010; Saputra, 2021; Indonesian National Disaster Management Agency, 2023), and one (2.1%) outlining risk factors to disaster-related deaths (Hu *et al.*, 2018). Details on reviewed studies can be found in the Supplementary Appendix 2.

Twenty (62.5%) studies (Gobel *et al.*, 2014; Indonesian Ministry of Health, 2015, 2017, 2020; Lesmana *et al.*, 2018; Sillehu and Kartika, 2018; Patimah, 2019; Patimah *et al.*, 2019; Hady *et al.*, 2020; Nugroho and Suryono, 2020; Ose *et al.*, 2020; Rosmi *et al.*, 2020; Suryono and Nugroho, 2020; Faradisi *et al.*, 2021; Nadapdap, 2021; Sugiantoro and Wahyudi, 2021; Sukarna *et al.*, 2021; Fernalia *et al.*, 2022; Pranoto *et al.*, 2023; Sadewa *et al.*, 2023) examined preventative aspects of unintentional drowning. Most of these studies (*n* = 19) described individual-focused approaches.

### Numbers and rates of unintentional drowning death in Indonesia

#### Accidental drowning deaths

Three descriptive observational studies (Usaputro and Yulianti, 2013; Wulur, 2013; Astreani and Alit, 2015) reported accidental drowning deaths at the subnational level, all outlining the epidemiological measures of drowning mortality as a count. The only data source for drowning deaths identified in these studies was medico-legal/autopsy records. Two of these studies (Usaputro and Yulianti, 2013; Astreani and Alit, 2015) reported drowning mortality at the provincial scale of Bali and included drowning deaths involving Indonesians and foreign nationals. Both studies reported drowning deaths documented by Sanglah Provincial Hospital in the province of Bali, with a total of 209 drowning deaths documented between 2010 and 2014, from which an average annual drowning mortality rate of 1.73/100 000 between 2010 and 2014 can be inferred. Meanwhile, one study (Wulur, 2013) reported 15 drowning deaths documented by the Forensic Department of Prof. Dr. R. D. Kandou Provincial Hospital of North Sulawesi between 2007 and 2011, from which an average annual drowning mortality of 0.18/100 000 can be inferred for the provincial level of North Sulawesi between 2007 and 2011. However, it is important to note that all drowning data reported in these studies were collected from provincial referral hospitals and may miss some incidents.

#### Disaster-related drowning deaths

Two papers (Hu *et al.*, 2018; Indonesian National Disaster Management Agency, 2023) in this review discussed disaster-related deaths. The Indonesian National Disaster Management Agency (2023) reported the frequency of disasters and disaster-related mortalities and missing cases in Indonesia between 1815 and 2023: (i) 13 927 flooding events, with 22 476 deaths and 8195 missing victims; (ii) 9503 flooding and avalanche events, with 3324 deaths and 379 missing victims; (iii) 499 tidal wave-related disaster events, with 165 deaths and 49 missing victims and (iv) 11 225 cyclone events, with 479 deaths and 49 missing victims. These hydrometeorological disasters, excluding tsunami, contributed to 36.7% (*N* = 100 434/259 407) of disaster-related deaths and 17% (*N* = 8672/51 037) of all disaster-related missing victims in Indonesia, from which disaster-related rate of 186 deaths per 100 000 disaster-affected populations can be inferred.

In a study using the Emergency Disasters Database (EM-DAT) and the Dartmouth Flood Observatory data to analyse floods and flood-induced mortality across the globe between 1975 and 2016, Hu *et al.* (2018) reported Indonesia among countries with the highest flood frequency and flood-induced mortality worldwide, estimating 5000 to 10 000 flood-induced deaths occurred in Indonesia between 1975 and 2016, with tropical cyclones contributing to 0–10% of these deaths, from which flood-induced rates of 1–3 deaths per 100 000 flood-affected populations can be inferred. However, it is important to note that the exact cause of the reported hydrometeorological disaster-related deaths, either by drowning or other causes, were not stated in these two studies, and that data for the period before 1990 may be severely underestimated. There was also no information available on exact data sources and how at-risk populations were estimated, hence the possibility of selection and measurement/information bias was identified in these studies, resulting in the difficulty of inferring cause-specific or proportionate mortality rates.

#### Water transport-related drowning deaths

Three studies reported water transport-related deaths in Indonesia (Suwardjo *et al.*, 2010; Saputra, 2021; Indonesian National Disaster Management Agency, 2023). The Indonesian National Disaster Management Agency (2023) reported that a total of 15 shipping/boating accidents occurred across Indonesia between 2014 and 2017, resulting in 121 deaths and 97 missing victims. Saputra (2021) reported on the completed investigations of 120 shipping accidents by the Indonesian National Transportation Safety Committee between 2003 and 2019, and found they contributed to 513 deaths and 701 missing victims across Indonesia. At the subnational level, Suwardjo *et al.* (2010) reported that 61 fishing vessel accidents occurred around the three subdistrict-level fishing ports of Central Java Province between 2006 and 2008, contributing to a total of 68 deaths, or an annual average of 32 dead/missing fishing crews, and an average fatality accident rate for all three study sites of 115 deaths per 100 000 fishermen, with 26.5% (*n* = 18/68) of victims falling off the ships into the water during shipping or fishing, and 45.6% (*n* = 31/68) of victims died due to the ship capsizing. However, it is important to note that the exact cause of the reported water transport-related deaths were not stated in these studies, and that these reports only included cases where the Indonesian Disaster Management Agency was involved in the rescue/evacuation process, or investigated by the Indonesian National Transportation Safety Committee, or reported by the authorities within the study site and period, hence it may miss some incidents and make inferring cause-specific or proportionate mortality rates difficult.

### Risk factors of fatal unintentional drowning in Indonesia

Risk factors were investigated in 11 studies, with seven studies reporting factors potentially contributing to accidental drowning events (Usaputro and Yulianti, 2013; Wulur, 2013; Astreani and Alit, 2015; Prasetyo, 2017; Widyastuti and Rustini, 2017; Elsi and Gusti, 2020; Welembuntu *et al.*, 2021), three discussing potential contributing factors to water transport-related drowning (Suwardjo *et al.*, 2010; Saputra, 2021; Indonesian National Disaster Management Agency, 2023), and one outlining risk factors to disaster-related drowning deaths (Hu *et al.*, 2018).

Several factors were identified as potential risk factors of for unintentional drowning in Indonesia: (i) sociodemographic characteristics, including age and sex (males aged 18 and over disproportionately contributed to the majority of drowning death cases in Bali and North Sulawesi (Usaputro and Yulianti, 2013; Wulur, 2013; Astreani and Alit, 2015)), nationality (foreign nationals and Indonesians made up for almost similar proportions of drowning deaths in Bali (Usaputro and Yulianti, 2013; Astreani and Alit, 2015), noting the high population of foreign nationals in the major tourist destination Bali may differ from populations of other provinces), population density (population density had a significantly positive correlation with the number of flood-related deaths (Hu *et al.*, 2018)), and income level (flood-induced mortality increased with the decrease of per capita GDP (Hu *et al.*, 2018)); (ii) environmental factors, including aquatic location of drowning deaths (most accidental and water transport-related drowning cases occurred in open seawater (Usaputro and Yulianti, 2013; Astreani and Alit, 2015; Indonesian National Disaster Management Agency, 2023)), seasonality (highest numbers of fishing vessel accidents were recorded during rainy seasons (Suwardjo *et al.*, 2010)), geographical and environmental conditions (a third of boating/shipping accidents occurred in Indonesia were weather-related (Indonesian National Disaster Management Agency, 2023), and most flooding inundation and flood-induced deaths occurred in low-lying regions with dense river systems (Hu *et al.*, 2018)), and hydrometeorological disasters (the frequency of floods and flood-induced mortality were generally increasing, with a large proportion of flood-induced deaths attributed to tropical cyclone-induced flash floods (Hu *et al.*, 2018)); (iii) risky behaviour (alcohol identified on a fifth of autopsied drowning victims, although there was no information on the blood alcohol content (Usaputro and Yulianti, 2013)); (iv) low knowledge and skills on water safety and water rescue, particularly knowledge on first aid for drowning victims (Prasetyo, 2017; Widyastuti and Rustini, 2017; Elsi and Gusti, 2020; Welembuntu *et al.*, 2021) and (v) low knowledge and compliance for boating, shipping, and maritime safety-related regulations, including poor maintenance of ships, lack of safety equipment on board, poor knowledge and compliance of safety regulations and underqualified seafarers and poor ship crews’ capacity in ensuring safe boating/maritime practice (Suwardjo *et al.*, 2010; Saputra, 2021; Indonesian National Disaster Management Agency, 2023), as summarized in **Table 1**.

**Table 1:** Studies on risk factors of unintentional drowning in Indonesia

Risk factors investigated		Study findings
Socio-demographic characteristic	Sex	• Male victims: 84.6% (*n* = 77/91), females: 15.4% (*n* = 14/91) of drowning deaths recorded by the Forensic Department of Sanglah Provincial Hospital of Bali from 2012 to 2014 (Astreani and Alit, 2015). • Male victims: 84.5% (*n* = 60/71), females: 15.5% (*n* = 11/71) of drowning deaths recorded by the Forensic Department of Sanglah Provincial Hospital of Bali between 2010 and 2012 (Usaputro and Yulianti, 2013). • Male victims: 80% (*n* = 12/15), females: 20% (*n* = 3/15) of drowning deaths recorded by the Forensic Department of Prof. Dr. R. D. Kandou General Hospital of North Sulawesi Province between 2007 and 2011 (Wulur, 2013). • No measures of association were reported, and a possibility of selection bias was identified in all three studies above (Usaputro and Yulianti, 2013; Wulur, 2013; Astreani and Alit, 2015).
	Age	• Adults: 87.9% (*n* = 80/91); children: 12.1% (*n* = 11/91) of drowning deaths in Bali from 2012 to 2014. The definition of the term ‘adult’ and ‘ children’ were not defined (Astreani and Alit, 2015). • Adults aged 21–30 years: 22.5% (*n* = 16/71), >50 years: 19.7% (*n* = 14/71), 31–40 years: 18.3% (*n* = 13/71) and <20 years: 16.9% (*n* = 12/71) of drowning deaths in Bali between 2010 and 2012 (Usaputro and Yulianti, 2013). • Adults aged ≥20 years: 86.7% (*n* = 13/15), children aged 5–14 years: 6.67% (*n* = 1/15) of drowning deaths in North Sulawesi Province between 2007 and 2011 (Wulur, 2013). • No measures of association were reported, and a possibility of selection and measurement/information bias was identified in all three studies above (Usaputro and Yulianti, 2013; Wulur, 2013; Astreani and Alit, 2015).
	Nationality	• Indonesians: 54.9% (*n* = 50/91), foreign nationals: 45.1% (*n* = 41/91) of drowning deaths in Bali between 2012 and 2014 (Astreani and Alit, 2015). • Foreign nationals: 49.3% (*n* = 35/71), Indonesians: 40.8% (*n* = 29/71) of drowning deaths in Bali between 2010 and 2012 (Usaputro and Yulianti, 2013). • No measures of association were reported, and a possibility of selection and measurement/information bias was identified in the two studies above (Usaputro and Yulianti, 2013; Astreani and Alit, 2015).
	Population density	• Significant positive correlation between population density and the number of flood-related deaths. A possibility of selection bias was identified (Hu *et al.*, 2018).
	Income level	• The flood-affected population and flood-induced mortality increased with the decrease of per capita GDP. A possibility of selection bias was identified (Hu *et al.*, 2018).
Environmental factor	Aquatic locations of drowning deaths	• Beaches: 69.2% (*n* = 63/91), swimming pools: 13.2% (*n* = 12/91), river: 13.2% (*n* = 12/91), bathroom and swamps: 4.4% (*n* = 4/91) of drowning deaths in Bali between 2012 and 2014 (Astreani and Alit, 2015). • Open seawater: 53.5% (*n* = 38/71), freshwater bodies: 25.4% (*n* = 18/71), unknown location: 21.1% (*n* = 15/71) of drowning deaths in Bali between 2010 and 2012 (Usaputro and Yulianti, 2013). • Open seas: 60% (*n* = 9/15), rivers (related to river crossings): 26.67% (*n* = 4/15), lakes and during flooding events: 6.67% (*n* = 1/15) of ship accidents between 2013 and 2017 (Indonesian National Disaster Management Agency, 2023). • No measures of association were reported, and a possibility of selection and measurement/information bias was identified in all three studies (Usaputro and Yulianti, 2013; Astreani and Alit, 2015; Indonesian National Disaster Management Agency, 2023).
	Seasonality	• Of 61 fishing vessel accidents reported to occur around three fishing ports of Central Java Province between 2006 and 2008, the highest numbers of fishing vessel accidents were recorded during rainy seasons (November to February). No measures of association were reported. A possibility of selection bias was identified (Suwardjo *et al.*, 2010).
	Geographical and environmental conditions	• Weather-related: 33.33% (*n* = 5/15) of ship accidents in Indonesia between 2014 and 2017. No measures of association were reported. A possibility of selection bias and measurement/information bias was identified (Indonesian National Disaster Management Agency, 2023). • Floodings and flood-induced deaths most often occurred in low-lying regions with dense river systems. Plain areas with slope of <0.5°: 40% of flood events and 50% of flood-induced deaths worldwide; plain areas with slopes 0.5°-15°: 60% of flood events and 48% of flood-induced deaths worldwide. No measures of association were reported. A possibility of selection bias was identified (Hu *et al.*, 2018).
	Hydrometeorological disasters	• Between 1975 and 2016, the frequency of floods and flood-induced mortality were generally increasing globally, including in Indonesia. • The annual variation of mortality per flood event was highly related to floods with higher intensity, with the highest flood frequency and flood-induced mortality worldwide reported to occur in Asia, particularly in China, India, Indonesia and the Philippines. • A large proportion of flood-induced deaths and the highest flood-induced mortality rate worldwide can be attributed to tropical cyclone-induced flash floods. • No measures of association were reported. A possibility of selection bias was identified in this study (Hu *et al.*, 2018).
Risky behaviours	Alcohol consumption	• Blood alcohol was identified in 20% (*n* = 4/20) of autopsied drowning victims at Sanglah Provincial Hospital of Bali between 2010 and 2012. No information on the blood alcohol content. No measures of association were reported (Usaputro and Yulianti, 2013).
Biological factors	Underlying medical conditions	• Comorbid conditions were recorded on 15% (*n* = 3/20) of drowning victims underwent autopsy at Sanglah Provincial Hospital of Bali between 2010 and 2012. No information on comorbid conditions. No measures of association were reported (Usaputro and Yulianti, 2013).
Knowledge and skills on water safety and water rescue	Knowledge on first aid for drowning victims	• Significant correlation between fishermen’s knowledge on Basic Life Support (BLS) and attitude to BLS being given for drowning victims (*p* < 0.05) (Elsi and Gusti, 2020). • Coastal area residents with ‘sufficient’ knowledge on first aid for drowning victims: 4.26% (*n* = 2/47), ‘insufficient’ level of knowledge: 87.23% (*n* = 41/47). No measures of association were reported (Prasetyo, 2017). • Coastal area residents with ‘sufficient’ level of knowledge on first aid for maritime accidents’ victims: 55% (*n* = 22/40), ‘good’ level of knowledge: 42.5% (*n* = 17/40). No measures of association were reported (Welembuntu *et al.*, 2021). • Coastal area residents with ‘sufficient’ level of knowledge on first aid for drowning victims: 57.14% (*n* = 20/35), ‘good’ level of knowledge: 31.4% (*n* = 11/35), ‘poor’ level of knowledge: 11.4% (*n* = 4/35). No measures of association were reported (Widyastuti and Rustini, 2017). • A possibility of selection bias and measurement bias was identified in all four studies above ((Prasetyo, 2017; Widyastuti and Rustini, 2017; Elsi and Gusti, 2020; Welembuntu *et al.*, 2021).
Water safety, safe boating and shipping, and maritime safety-related regulations	Types of boat/ship	• Passenger boats: 86.67% (n/13/15), fishing boat: 6.67% (*n* = 1/15), rescue boat: 6.67% (*n* = 1/15) of ship accidents in Indonesia between 2014 and 2017 (Indonesian National Disaster Management Agency, 2023). • Motorboats including cargo ships, bulk carriers, container ships and passenger ships (ferries and Ro-Ro ferries): 89% (*n* = 107/120) of ship accidents investigated by the Indonesian National Transportation Safety Committee between 2003 and 2019 (Saputra, 2021). • No measures of association were reported, and a possibility of selection and measurement/information bias was identified in two studies above (Indonesian National Disaster Management Agency, 2023; Saputra, 2021).
	Knowledge and compliance to regulations on water safety, safe boating and shipping, and maritime safety	• Boat overloading: 33.33% (*n* = 5/15), collisions: 13.33% (*n* = 2/15) of ship accidents in Indonesia between 2014 and 2017 (Indonesian National Disaster Management Agency, 2023). • Fire: 37% (*n* = 44/120); submersion/sinking: 28% (*n* = 34/120), collisions: 18% (*n* = 22/120), other causes: 17% (*n* = 20/120) of ship accidents investigated by the Indonesian National Transportation Safety Committee between 2003 and 2019. Contributing factors to shipping accidents identified were (i) poor maintenance of ships; (ii) unavailability/poor maintenance of safety equipment on board; (iii) poor knowledge, awareness and compliance of safety regulations and (iv) underqualified seafarers and poor ship crews’ capacity in ensuring safe shipping/maritime practice (Saputra, 2021). • Contributing factors to fishing vessel accidents in Central Java Province between 2006 and 2008: (i) underqualified shipping crews; (ii) poor knowledge, awareness, and compliance of safety regulations; (iii) unfulfillment of safety requirements. Approximately 84.3% of skippers and ship crews did not go beyond primary level education, hence did not qualify to undertake Basic Safety Training for shipping crews. Seventy per cent (*n* = 45/64) of all fishing vessels registered did not fulfil safety requirements due to insufficient number of life jackets and rescue buoys, unequipped with fire extinguishers and life rafts, and lacking in other safety equipment (Suwardjo *et al.*, 2010). • No measures of association were reported, and a possibility of selection and measurement/information bias was identified in all three studies above in all three studies above (Suwardjo *et al.*, 2010; Saputra, 2021; Indonesian National Disaster Management Agency, 2023).

However, it is important to note that despite the importance of providing an understanding of risk factors for drowning across Indonesia, no relative risk (RR) or odd ratio (OR) was reported across all studies reviewed, and no studies reported statistical correlations of risk factors of interest in association to fatal unintentional drowning incidents. All information on unintentional drowning risk factors was presented as proportions/counts of drowning deaths between categories of risk factors (**Table 1**).

### Drowning prevention in Indonesia

Analysis of the application of the Health Promotion Framework (Talbot and Verrinder, 2017) across 16 peer-reviewed papers (Gobel *et al.*, 2014; Lesmana *et al.*, 2018; Sillehu and Kartika, 2018; Patimah, 2019; Patimah *et al.*, 2019; Hady *et al.*, 2020; Nugroho and Suryono, 2020; Ose *et al.*, 2020; Rosmi *et al.*, 2020; Suryono and Nugroho, 2020; Faradisi *et al.*, 2021; Sugiantoro and Wahyudi, 2021; Sukarna *et al.*, 2021; Fernalia *et al.*, 2022; Pranoto *et al.*, 2023; Sadewa *et al.*, 2023) and four grey literature sources (Indonesian Ministry of Health, 2015, 2017, 2020; Nadapdap, 2021) on drowning prevention interventions revealed that most prevention approaches were midstream and downstream individual-focused, behaviour-based intervention, with a focus on education to build knowledge and skills, as outlined on **Table 2**. The interventions included providing health information/education on (i) emergency first aid for drowning victims (Gobel *et al.*, 2014; Lesmana *et al.*, 2018; Patimah, 2019; Patimah *et al.*, 2019; Hady *et al.*, 2020; Nugroho and Suryono, 2020; Ose *et al.*, 2020; Suryono and Nugroho, 2020; Faradisi *et al.*, 2021; Nadapdap, 2021; Sugiantoro and Wahyudi, 2021; Sukarna *et al.*, 2021; Fernalia *et al.*, 2022; Pranoto *et al.*, 2023); (ii) water rescue (Sillehu and Kartika, 2018; Rosmi *et al.*, 2020; Sukarna *et al.*, 2021; Sadewa *et al.*, 2023) and (iii) drowning prevention awareness (Indonesian Ministry of Health, 2015, 2020). These educational interventions were aimed at diverse populations, including local street stallholders in coastal areas, fishermen, youth groups, tourism awareness group members and community health workers.

**Table 2:** Analysis of prevention interventions investigated in Indonesia using the Health Promotion Framework

Author	Type of publication	Population investigated	Study design	Intervention method investigated	Prevention aspect investigated in relation to Health Promotion Framework			Relevant findings
					Medical/ Individual approach	Behavioural approach	Socio-environmental approach	
Faradisi *et al.* (2021)	Original research article	Twenty-one street stallholders on Nyamplung Beach, Central Java Province	Pre-test, post-test study	Health education on Cardio-Pulmonary Resuscitation (CPR) on drowning victims	-	HE^1^	-	• Proportion of participants with good level of knowledge: post-intervention > pre-intervention. • No measures of association were reported. • A possibility of selection bias and measurement bias was identified.
Fernalia *et al.* (2022)	Original research article	Community members at Lingkar Barat Subdistrict, Bengkulu Province (data on the number of participants are not available)	Community service project	Health education on performing water rescue.	-	HE	CA	• Water rescue-trained local community group was formed. • No evaluation on participants’ level of knowledge and skills on water rescue. • No measures of association were reported. • A possibility of selection bias and measurement bias was identified.
Gobel *et al.* (2014)	Original research article	Forty-seven fishermen from a coastal area of North Bolaang Mongondow Regency, North Sulawesi Province	Pre-test, post-test study	Health education on first aid for drowning victims	-	HE	-	• Significant increase on the mean level of knowledge after the intervention applied (*p*<0.05). • No information on long-term knowledge retention. • A possibility of selection bias and measurement bias was identified.
Hady *et al.* (2020)	Original research article	Fifty residents of a coastal area of Takalar Regency, South Sulawesi Province	Pre-test, post-test study	Health education and roleplay on first aid for drowning victims	-	HE	-	• Significant increase on the mean level of knowledge after the intervention applied (*p*<0.05). • No information on long-term knowledge retention. • A possibility of selection bias and measurement bias was identified.
Indonesian Ministry of Health (2020)	Grey literature (government report)	NA	NA	Development of the National Drowning Prevention Strategy and Coordinating Agency; Development of drowning prevention awareness campaign for school-age children	-	SM	PD, RA	• The Directorate of Occupational and Sports Health (of the Indonesian Ministry of Health) will initiate four aspects of drowning prevention on national level: a) Development of the National Drowning Prevention Coordinating Agency b) Development of coordinated mechanisms of disseminating and monitoring health data c) Development of the National Drowning Prevention Strategy d) Development of drowning prevention awareness campaign targeting school-age children across Indonesia. • Indonesian Ministry of Health will initiate a multisectoral coordination with the Indonesian Coordinating Ministry for Maritime and Investment Affairs; Ministry of Marine Affairs and Fisheries; Ministry of Youth and Sports Affairs; Ministry of Transportation; Ministry of Education and Culture; Ministry of Tourism; National Bureau of Statistics; Maritime Security Agency; Sea and Coast Guard; National Search and Rescue Agency; National Agency for Disaster Management; National Police, Seafarers Union; and Maritime Doctors Association.
Indonesian Ministry of Health (2017)	Grey literature (policy statement of clinical standard)	NA	NA	Health information on pre-hospital and intrahospital emergency care for trauma patients	HI	-	-	• National clinical practice guideline for treatment of trauma cases, including for drowning victims. • Targeting Indonesian medical doctors.
Indonesian Ministry of Health (2015)	Grey literature (policy statement)	NA	NA	Health information on preventing drowning in children	HI	-	-	• Handbook on preventing drowning in children. • Targeting health workers and trained community members.
Lesmana *et al.* (2018)	Original research article	Forty-six respondents living in a coastal area of Amal Beach, Tarakan City, North Kalimantan Province	Pre-test, post-test study	Health education on first aid for drowning victims	-	HE	-	• Proportion of participants with good level of knowledge: post-intervention > pre-intervention. • No measures of association were reported. • A possibility of selection bias and measurement bias was identified.
Nadapdap (2021)	Grey literature (thesis)	Systematic search on 7 national journals and three international journals, using keywords “knowledge”, “first aid”, “drowning” and “basic life support”	Literature review	Health education on Basic Life Support (BLS) for drowning victims	-	HE	-	• Review identified respondents’ knowledge as ‘good’ after being given health education on BLS for drowning victims. • A possibility of selection bias and measurement bias was identified.
Nugroho and Suryono (2020)	Original research article	Fifteen members of a local freshwater fishing community of Kediri Regency, East Java Province	Pre-test, post-test study	Health education on first aid for infant drowning victims	-	HE	-	• Significant increase of the mean level of self-efficacy in emergency handling of toddler drowning victims after the intervention applied (*p*<0.05). • No information on long-term knowledge and self-efficacy retention. • A possibility of selection bias and measurement bias was identified.
Ose *et al.* (2020)	Original research article	Thirty-two volunteer village health workers overseeing a coastal area of Amal Beach, Tarakan City, North Kalimantan Province	Pre-test, post-test study	Health education on performing CPR on drowning victims	-	HE	-	• Significant increase of the mean level of knowledge after the intervention applied (*p*<0.05). • No information on long-term knowledge retention. • A possibility of selection bias and measurement bias was identified.
Patimah (2019)	Original research article	Eighteen residents of a coastal area of Jayapura City, Papua Province	Pre-test, post-test study	Health education on BLS and evacuation for drowning victims	-	HE	-	• Significant increase of the mean level of knowledge after the intervention applied (*p*<0.05). • No information on long-term knowledge retention. • A possibility of selection bias and measurement bias was identified.
Patimah *et al.* (2019)	Original research article	Fifty-eight residents of a coastal area of Jayapura District, Papua	Pre-test, post-test study	Health education on first aid for drowning victims	-	HE	-	• Proportion of participants with good level of knowledge: post-intervention > pre-intervention. • No measures of association were reported. • A possibility of selection bias and measurement bias was identified.
Pranoto *et al.* (2023)	Original research article	Sixty members of tourism awareness groups on Mentawai Islands, West Sumatra Province	Community service project	Health education on performing water rescue and CPR on drowning victims	-	HE	-	• No evaluation on participants’ level of knowledge and skills of on water rescue and CPR. • No measures of association were reported. • A possibility of selection bias and measurement bias was identified.
Rosmi *et al.* (2020)	Original research article	Thirty-six members of youth organizations (Karang Taruna) on Gresik Regency, East Java Province	Community service project	Health education on the dangers of flooding and performing water rescue on flooding-related drowning victims	-	HE	CA	• Water rescue-trained local community group was formed. • No evaluation on participants’ level of knowledge and skills on flooding-related water rescue. • No measures of association were reported. • A possibility of selection bias and measurement bias was identified.
Sadewa *et al.* (2023)	Original research article	Thirty-five participants consisting of lifeguards, pool attendants, swimming trainers, students, and swimming pool visitors at the Faculty of Sports and Health Sciences, Yogyakarta State University, Yogyakarta Province	Descriptive quantitative and qualitative approach	The development of modified water rescue tools (jerry cans lined with Styrofoam and equipped with ropes) for water rescue efforts, as an effective, cheap, easy to obtain substitute to water rescue equipment	HI	-	-	• The modified water rescue tool was deemed “very decent” by participants to be utilized on water rescue efforts. • No evaluation on the implementation and outcome of the intervention. • A possibility of selection bias and measurement bias was identified.
Sillehu and Kartika (2018)	Original research article	Thirty-five regency-level National Search and Rescue Agency staff members for Ambon City, Maluku Province	Analytical observational	Water rescue practice	HI	-	-	• Significant correlation between the performance by the National Search and Rescue Agency and the rescue of drowning victims (*p*<0.05). • A possibility of selection bias and measurement bias was identified.
Sugiantoro and Wahyudi (2021)	Original research article	Fifteen fishermen from Lempasing Regency, Lampung Province	Pre-test, post-test study	Health information on first aid for drowning victims	HI	-	-	• Significant increase of the mean level of knowledge and attitude after the intervention applied (*p*<0.05). • No information on long-term knowledge retention. • A possibility of selection bias and measurement bias was identified.
Sukarna *et al.* (2021)	Original research article	Thirty street stallholders on a coastal area on the province of Bangka Belitung Islands	Pre-test, post-test study	Health education on performing CPR and evacuation on drowning victims	-	HE	-	• Significant increase of the mean level of knowledge after the intervention applied (*p*<0.05). • No information on long-term knowledge retention. • A possibility of selection bias and measurement bias was identified.
Suryono and Nugroho (2020)	Original research article	Fifteen members of a local freshwater fishing community of Kediri Regency, East Java Province	Pre-test, post-test study	Health education on first aid for infant drowning victims	-	HE	-	• Proportion of participants with sufficient level of knowledge: post-intervention > pre-intervention. • No measures of association were reported. • A possibility of selection bias and measurement bias was identified.

CA: Community action; HE: Health education; HI: Health information; PD: Policy development; RA: Regulatory activity; SD: Skills development; SM: Social marketing.

Only one grey literature source (Indonesian Ministry of Health, 2020) reported on regulatory activities and social marketing approaches for drowning prevention. Meanwhile, two studies (Rosmi *et al.*, 2020; Fernalia *et al.*, 2022) outlined the potential of training local community groups to perform and assist on local water rescue efforts (**Table 2**).

## DISCUSSION

This scoping review aimed to describe the epidemiology of and risk factors for unintentional drowning in Indonesia and explore existing health promotion and prevention approaches currently in place. Despite a dire need to provide better understanding on the magnitude of drowning as a health problem in Indonesia, limited literature reporting on drowning epidemiology, risk factors or prevention and health promotion approaches in Indonesia were identified.

### Drowning mortality data across Indonesia: the disparity on data availability

Comprehensive data are imperative for agenda setting (World Health Organization, 2017). However, this review outlined the limited availability of drowning data across Indonesia. The data sources for drowning deaths identified in this review were derived from medico-legal autopsy records, records of investigated shipping accidents and rescue reports, with no drowning data derived from health and demographic surveillance data, national death registry, integrated coronial information system, police department records, national health reports or news reports. This is due to the unavailability of a coordinated national drowning data collection system and national death registry in Indonesia, from which national and subnational data for all categories of unintentional drowning can be recorded (World Health Organization, 2021). This unavailability underlines the possibility of under-representation of drowning data and a public health issue regarding the health system capacity on collecting, reporting and utilizing health data.

A similar reliance on medico-legal reports has been reported in other LMICs, such as in South Africa, where the dependence on hospital-based reports means that the surveillance system may miss drowning-related injuries which occur in and around homes, particularly in rural areas with inadequate access to healthcare facilities (Matzopoulos, 2002; Donson and Van Niekerk, 2013; Morris *et al.*, 2016). The underreporting of drowning incidents can be linked to the nature of drowning deaths, where victims often suffer a quick death on location and never reach medical facilities or are reported to authorities, which may impede accurate data collection in countries with dependence on facility-based reporting systems, such as in Indonesia (Linnan *et al.*, 2012, 2013; World Health Organization, 2014). These limitations underline the urgent need to strengthen public health system capacity; develop standardized, national health, death and coronial data reporting frameworks; enhance multi-sectoral collaboration and advocate for political and financial investment, to develop a robust drowning data collection system in Indonesia (Schuurman *et al.*, 2011; Reynolds *et al.*, 2013; Scarr and Jagnoor, 2022).

### Drowning risk factors in Indonesia: a knowledge gap

This review identified a lack of exploration on predictors for unintentional drowning fatalities, with no studies reporting RR/OR or statistical correlations of predictors of interest and fatal unintentional drowning incidents. This may hinder the development of prevention interventions needed across different provinces of Indonesia, which may be different to prevention interventions which have been shown to be effective in high income countries (Bennett and Linnan, 2014; Franklin and Scarr, 2014; Hyder *et al.*, 2014; Linnan *et al.*, 2014).

Increasing supervision of children, raising community awareness and skills on water safety, and encouraging community participation have been identified as important protective factors for drowning in LMICs (Rahman *et al.*, 2010, 2012; Cenderadewi *et al.*, 2020). As advocated by the WHO, the effectiveness of community driven crèches in preventing child drowning for children aged 12–59 months, and swimming lessons in reducing the risk of drowning for children aged 6 and above, along with their cost effectiveness, have been widely reported in rural settings in Bangladesh (Rahman *et al.*, 2012; Talab *et al.*, 2016; Alonge *et al.*, 2020; Alfonso *et al.*, 2021; Rahman and Rahman, 2022). Further scaling up of these interventions to other LMICs, including to Indonesia, should be considered, as part of the global effort to reduce the global burden of drowning, particularly among children (Rahman *et al.*, 2012). Further research is required to evaluate the process, impact and outcome of the interventions against key indicators in Indonesia; improving local capacity and understanding in preventing drowning in the country (Guevarra *et al.*, 2021).

Further research on contributing factors to disaster-related and water transport-related drowning deaths is also imperative to inform the development of drowning prevention approaches suitable for Indonesian context, given the high frequency of hydrometeorological disasters and water transport-related incidents and the nation’s high vulnerability to the impacts of disasters and climate change (Suwardjo *et al.*, 2010; Muis *et al.*, 2015; Djalante and Garschagen, 2017; Hu *et al.*, 2018; Sari and Prayoga, 2018; Mantong *et al.*, 2020; Saputra, 2021; Indonesian National Disaster Management Agency, 2023a, 2023b).

### Understanding the linkage between drowning prevention and health promotion

Using the Health Promotion Framework (Talbot and Verrinder, 2017) to analyse the socio-ecological approaches utilized in the prevention of drowning in Indonesia, this review identified the under-exploration of the concept of the socio-ecological approach of health promotion related to drowning prevention in Indonesia, leaving the research area of community participation and development of evidence-informed policy around water safety relatively neglected. The limited availability of population-focused midstream and upstream drowning prevention interventions in Indonesia does not align with the underlying premise of the Health Promotion Framework, which supports the need for multisectoral, multi-strategic approaches including educational, behavioural, socio-environmental and regulatory approaches to ensure effective individual and community-level injury prevention (Stempski *et al.*, 2015; Denehy *et al.*, 2016; Leavy *et al.*, 2016; Giles *et al.*, 2017; World Health Organization Regional Office for the Eastern Meditteranean, 2017). A comprehensive approach to drowning prevention in Indonesia requires approaches that go across the downstream, midstream, upstream continuum and include strengthening individual and broader community level knowledge and skills, fostering coalitions and networks, changing organizational practices and policy and legislation setting (Franklin and Scarr, 2014).

### Recommendations: highlights for policy development, practice and future research

#### Policy development and practice

A national, evidence-informed regulatory framework for drowning prevention, guided by the WHO implementation guide on preventing drowning (World Health Organization, 2017) and affirmed by the 2021 United Nations General Assembly Resolution on global drowning prevention (United Nations, 2021), is required to reduce drowning fatalities in Indonesia. To inform this, a situational assessment must be performed in Indonesia, to: (i) review available data on drowning; (ii) assess current efforts regarding drowning prevention including existing policy and regulation; (iii) identify key stakeholders who play a role in drowning prevention and (iv) assess required human and financial resources (World Health Organization, 2017). This approach is supported by the WHO, who also recommends advancing drowning prevention through robust data collection, to inform burden and risk factor identification, and agenda setting, as well as development and evaluation of regulatory frameworks and prevention interventions (World Health Organization, 2017).

#### Contributing to situational assessment on drowning prevention in Indonesia

Several findings of this review may inform the situational assessment on drowning prevention in Indonesia. Furthermore, although not included in this study, several studies identified during the database searches may provide baseline information around disaster risk management and enforcement of maritime, shipping and boating safety in Indonesia, informing the urgently needed multisectoral collaboration to develop coordinated national drowning prevention efforts:

1 The unavailability of coordinated national death registry in Indonesia, from which national and subnational subcategory drowning data can be collected, underlines the possibility of under-representation of drowning data. Developing a robust and consolidated drowning data collection system, which employs data triangulation methodology, combining data from national death and coronial registry, organizational reports and media report monitoring, is imperative to inform the drowning prevention agenda in Indonesia.
2 The Indonesian Ministry of Health (2020) initiated the development of the National Drowning Prevention Strategy and Coordinating Agency, in conjunction with the strengthening of health data dissemination and monitoring, and development of social marketing approaches to increase community awareness on drowning prevention. This initiative was undertaken as a response to the WHO call for member nations to strengthen their national drowning data collection (World Health Organization, 2021). Main actors for drowning prevention include the Indonesian Ministry of Health as the lead agency, in coordination with other relevant agencies (outlined in **Table 2**) (Indonesian Ministry of Health, 2020). Further research is needed to update the progress of the initiative and evaluate the impact and outcome of interventions in reducing drowning against key indicators.
3 Main actors on disaster risk management include the National Disaster Management Agency as the lead agency; Meteorological, Climatological, and Geophysical Agency; Ministry of Public Works; Ministry of Environment and Forestry; Spatial Planning Agencies; river basin management authorities; National Search and Rescue Agency; Red Cross Society; and National Police and Military Forces. Limited budget (only 0.1–0.4% of national budgeting were allocated for disaster risk reduction efforts at the local level), low human resource capacity, non-integrated regulations and unclear coordination mechanisms across government agencies were the apparent challenges (Muis *et al.*, 2015; Hapsari and Zenurianto, 2016; Buchori *et al.*, 2018; Handayani *et al.*, 2019; Isa *et al.*, 2019).
4 Main actors on maritime and boating safety enforcement include the Ministry of Transportation as the lead agency, harbourmasters and Sea and Coast Guard. Low human resource capacity, low compliance to regulations, overlapping regulatory frameworks with unclear responsibilities and coordination mechanisms between agencies and the geographic nature of Indonesia as a vast archipelagic nation were identified as challenges in enforcing maritime and boating safety nationwide (Andry and Yuliani, 2014; Mantong *et al.*, 2020).
5 A continuous effort to systemically integrate drowning prevention framework across regulatory activities enforcing water safety, adequate supply of clean water, maritime/boating safety, occupational health and safety, disaster risk management and sustainable spatial and non-spatial development planning, emphasizing the importance of disaster risk reduction and climate change adaptation to the efforts of reducing fatalities, with clear obligations, responsibilities and coordination mechanisms between agencies, is required in Indonesia.

### Future research

More research on drowning is needed in Indonesia across all domains, however several priorities for future research were noted, with a particular focus on developing and improving the performance of drowning surveillance to ensure the availability and reliability of drowning mortality and morbidity data across Indonesia. Studies investigating the measurement of association between accidental, disaster- and water transport-related drowning risk factors, and the incidence of fatal and non-fatal unintentional drowning incidents are essential to inform the appropriate prevention measures for Indonesia. Further research on drowning cases related to disasters, disabilities, genders, older populations and children is also vital to provide a better understanding of the at-risk populations in the country (Pearn and Franklin, 2013; Franklin *et al.*, 2014; Peden and Franklin, 2019; Clemens *et al.*, 2021; Roberts *et al.*, 2021).

In addition, further investigation on broader health promotion approaches that reflect a socio-ecological approach to drowning prevention is imperative for Indonesian context. It is particularly important to explore the potential vital link between unintentional drowning prevention and safe water provision, boating and maritime safety regulations and enforcement, occupational safety and health, rural development, disaster risk management and efforts to bridge economic inequities and disparities in accessing healthcare across different populations, particularly impacting the socioeconomically disadvantaged populations of rural and remote areas of archipelagic Indonesia (Suwardjo *et al.*, 2010; Muis *et al.*, 2015; Djalante and Garschagen, 2017; Hu *et al.*, 2018; Sari and Prayoga, 2018; Mantong *et al.*, 2020; Saputra, 2021; Indonesian National Disaster Management Agency, 2023a, 2023b).

### Strengths and limitations

Several factors contributed to the strength of this review, including the inclusion of studies published in Indonesian language and the use of broad search terms to capture the extensive causes of drowning and the wide areas where drowning may occur. However, several limitations were also identified. Firstly, it is possible that not all studies exploring unintentional drowning in Indonesia were located. In addition, the lack of information on accidental, disaster-related and water transport-related drowning mortality numbers, cause of deaths, data sources and at-risk populations, along with the inconsistency in data collection and reporting between articles, may compromise the accuracy and quality of drowning data identified, limiting the generalizability and mortality rate inference from the findings, potentially resulting in the underestimation of the magnitude of unintentional drowning mortality across Indonesia.

## CONCLUSION

Limited publications on drowning rates, risk factors and prevention were observed within Indonesia. The unavailability of a coordinated national drowning data collection system in Indonesia underlines the possibility of under-representation of drowning mortality. The under-investigated measurement of association between various exposures and fatal drowning incidents was identified, potentially hindering the development of prevention interventions. The over-reliance on individual-focused prevention measures was observed in Indonesia, with an apparent under-development of socio-ecological health promotion approaches to drowning prevention. Several highlights for future research were noted, with a particular focus on improving the performance of drowning surveillance, to ensure the availability and reliability of drowning mortality and morbidity data across Indonesia. Broader health promotion approaches that reflect a socio-ecological approach to drowning prevention in Indonesia is also imperative in reducing drowning incidents and fatalities in Indonesia.

## Supplementary Material

daad130_suppl_Supplementary_Appendix
