# Supplementary material for: Fatal drowning in Indonesia: understanding knowledge gaps through a scoping review
Source: Health Promot Int. 2023 Oct 18;38(5):daad130. doi: 10.1093/heapro/daad130 (PMC10583758; doi:10.1093/heapro/daad130)
Supplement: daad130_suppl_Supplementary_Appendix [file daad130_suppl_supplementary_appendix.docx]

## Appendix 1. Search strings for each database

| Databases | Language of publications | Search strings |
| --- | --- | --- |
| MEDLINE (Ovid) | English | (drown*) AND indonesia |
|  |  | exp Drowning/ AND indonesia.mp. |
|  |  | (swim*) AND indonesia |
|  |  | swimming/ AND indonesia.mp |
|  |  | maritime AND indonesia |
|  |  | (flood*) AND indonesia |
|  |  | exp Floods/ AND indonesia.mp |
|  |  | ((hurricane*) OR (cyclone*) OR (typhoon*) OR (monsoon*)) AND indonesia |
|  |  | exp Cyclonic Storms/ and exp Indonesia/ |
|  |  | (disaster*) AND indonesia |
|  |  | (exp Disasters/ or exp Natural Disasters/) AND exp Indonesia/ |
|  |  | (water rescue) AND indonesia |
|  |  | ((boat*) OR (ship*)) AND indonesia |
|  |  | exp Ships/ and *Indonesia/ |
|  | Indonesian | tenggelam |
|  |  | renang OR berenang |
|  |  | banjir OR badai OR bencana |
|  |  | kecelakaan AND (kapal OR perkapalan OR layar OR pelayaran OR laut) |
|  |  | Keselamatan AND (kapal OR perkapalan OR layar OR pelayaran OR laut) |
| CINAHL | English | (drown*) AND indonesia |
|  |  | TX drown* AND indonesia |
|  |  | (MH "Drowning+") AND (MM "Indonesia") |
|  |  | (swim*) AND indonesia |
|  |  | (MM "Swimming") AND (MM "Indonesia") |
|  |  | maritime AND indonesia |
|  |  | (flood*) AND indonesia |
|  |  | ((hurricane*) OR (cyclone*) OR (typhoon*) OR (monsoon*)) AND indonesia |
|  |  | (MM "Natural Disasters+") AND (MM "Indonesia") |
|  |  | (MM "Water Rescue") AND (MM "Indonesia") |
|  |  | ((boat*) OR (ship*)) AND indonesia |
|  |  | (MH "Ships+") AND (MH "Indonesia") |
|  | Indonesian | tenggelam |
|  |  | renang OR berenang |
|  |  | banjir OR badai OR bencana |
|  |  | penyelamatan AND (air OR perairan) |
|  |  | kecelakaan AND (kapal OR perkapalan OR layar OR pelayaran OR laut) |
|  |  | keselamatan AND (kapal OR perkapalan OR layar OR pelayaran OR laut) |
| Informit | English | (drown*) AND indonesia |
|  |  | (swim*) AND indonesia |
|  |  | maritime AND indonesia |
|  |  | (flood*) AND indonesia |
|  |  | ((hurricane*) OR (cyclone*) OR (typhoon*) OR (monsoon*)) AND indonesia |
|  |  | (disaster*) AND indonesia |
|  |  | (water rescue) AND indonesia |
|  |  | ((boat*) OR (ship*)) AND indonesia |
|  | Indonesian | tenggelam |
|  |  | renang OR berenang |
|  |  | banjir OR badai OR bencana |
|  |  | penyelamatan AND (air OR perairan) |
|  |  | kecelakaan AND (kapal OR perkapalan OR layar OR pelayaran OR laut) |
|  |  | keselamatan AND (kapal OR perkapalan OR layar OR pelayaran OR laut) |
| PsycINFO (ProQuest) | English | (drown*) AND indonesia |
|  |  | (swim*) AND indonesia |
|  |  | maritime AND indonesia |
|  |  | (flood*) AND indonesia |
|  |  | ((hurricane*) OR (cyclone*) OR (typhoon*) OR (monsoon*)) AND indonesia |
|  |  | (disaster*) AND indonesia |
|  |  | (water rescue) AND indonesia |
|  |  | ((boat*) OR (ship*)) AND indonesia |
|  | Indonesian | tenggelam |
|  |  | renang OR berenang |
|  |  | banjir OR badai OR bencana |
|  |  | kecelakaan AND (kapal OR perkapalan OR layar OR pelayaran OR laut) |
|  |  | keselamatan AND (kapal OR perkapalan OR layar OR pelayaran OR laut) |
| Scopus | English | (drown*) AND indonesia |
|  |  | (swim*) AND indonesia |
|  |  | (maritime safety) AND indonesia |
|  |  | (flood*) AND indonesia |
|  |  | ((hurricane*) OR (cyclone*) OR (typhoon*) OR (monsoon*)) AND indonesia |
|  |  | (disaster*) AND indonesia |
|  |  | (water rescue) AND indonesia |
|  |  | ((boat*) OR (ship*)) AND indonesia |
|  | Indonesian | tenggelam |
|  |  | renang OR berenang |
|  |  | banjir OR badai OR bencana |
|  |  | penyelamatan AND (air OR perairan) |
|  |  | kecelakaan AND (kapal OR perkapalan OR layar OR pelayaran OR laut) |
|  |  | keselamatan AND (kapal OR perkapalan OR layar OR pelayaran OR laut) |
| SafetyLit | English | (drown*) AND Indonesia |
|  |  | (swim*) AND indonesia |
|  |  | maritime AND indonesia |
|  |  | (flood*) AND indonesia |
|  |  | ((hurricane*) OR (cyclone*) OR (typhoon*) OR (monsoon*)) AND indonesia |
|  |  | (disaster*) AND indonesia |
|  |  | (water rescue) AND indonesia |
|  |  | ((boat*) OR (ship*)) AND indonesia |
|  | Indonesian | tenggelam |
|  |  | renang OR berenang |
|  |  | banjir OR badai OR bencana |
|  |  | penyelamatan AND (air OR perairan) |
|  |  | kecelakaan AND (kapal OR perkapalan OR layar OR pelayaran OR laut) |
|  |  | Keselamatan AND (kapal OR perkapalan OR layar OR pelayaran OR laut) |
| BioMed Central | English | (drown*) AND Indonesia |
|  |  | (swim*) AND indonesia |
|  |  | (maritime safety) AND indonesia |
|  |  | (flood*) AND indonesia |
|  |  | ((hurricane*) OR (cyclone*) OR (typhoon*) OR (monsoon*)) AND indonesia |
|  |  | (disaster*) AND indonesia |
|  |  | (water rescue) AND indonesia |
|  |  | ((boat*) OR (ship*)) AND indonesia |
|  | Indonesian | tenggelam |
|  |  | renang OR berenang |
|  |  | banjir OR badai OR bencana |
|  |  | kecelakaan AND (kapal OR perkapalan OR layar OR pelayaran OR laut) |
|  |  | keselamatan AND (kapal OR perkapalan OR layar OR pelayaran OR laut OR maritim) |
| Google Scholar | English | allintitle: (drown OR drowns OR drowned OR drowning) indonesia |
|  |  | allintitle: (swim OR swims OR swimming) indonesia |
|  |  | allintitle: (maritime safety) indonesia |
|  |  | allintitle: (flood OR floods OR flooding) indonesia |
|  |  | allintitle: (hurricane OR hurricanes OR cyclone OR cyclones OR typhoon OR typhoons OR monsoon OR monsoons) indonesia |
|  |  | allintitle: (disaster OR disasters) (drowning OR drowned OR drowns OR drown) indonesia |
|  |  | allintitle: (water rescue) indonesia |
|  |  | allintitle: (boat OR boats OR boating OR ship OR ships OR shipping) indonesia |
|  | Indonesian | allintitle: tenggelam |
|  |  | allintitle: renang OR berenang |
|  |  | allintitle: (banjir OR badai OR bencana) tenggelam |
|  |  | allintitle: (air OR perairan) penyelamatan |
|  |  | allintitle: kecelakaan (kapal OR perkapalan OR layar OR pelayaran OR laut) |
|  |  | allintitle: keselamatan (kapal OR perkapalan OR layar OR pelayaran OR laut OR maritim) |
| Sinta | Indonesian | tenggelam |
|  |  | renang |
|  |  | berenang |
|  |  | banjir tenggelam |
|  |  | badai tenggelam |
|  |  | bencana tenggelam |
|  |  | kecelakaan kapal |
|  |  | kecelakaan layar |
|  |  | kecelakaan pelayaran |
|  |  | kecelakaan laut |
|  |  | keselamatan kapal |
|  |  | keselamatan perkapalan |
|  |  | keselamatan layar |
|  |  | keselamatan pelayaran |
|  |  | keselamatan laut |
|  |  | keselamatan maritim |
|  |  | water rescue |
|  |  | penyelamatan air |
|  |  | Penyelamatan perairan |
| Garuda | Indonesian | tenggelam |
|  |  | renang |
|  |  | berenang |
|  |  | banjir tenggelam |
|  |  | badai tenggelam |
|  |  | bencana tenggelam |
|  |  | kecelakaan kapal |
|  |  | kecelakaan layar |
|  |  | kecelakaan pelayaran |
|  |  | kecelakaan laut |
|  |  | keselamatan kapal |
|  |  | keselamatan perkapalan |
|  |  | keselamatan layar |
|  |  | keselamatan pelayaran |
|  |  | keselamatan laut |
|  |  | keselamatan maritim |
|  |  | water rescue |
|  |  | penyelamatan air |
|  |  | penyelamatan perairan |
| Government websites  (Google) |  | tenggelam site:.go.id |
|  |  | tenggelam site:.org |
|  |  | kematian tenggelam site:.go.id |
|  |  | kematian tenggelam site:.org |
|  |  | faktor resiko tenggelam site:.go.id |
|  |  | faktor resiko tenggelam site:.org |
|  |  | pencegahan tenggelam site:.go.id |
|  |  | pencegahan tenggelam site:.org |
|  |  | renang site:.go.id |
|  |  | renang site:.org |
|  |  | berenang site:.go.id |
|  |  | berenang site:.org |
|  |  | banjir OR badai OR bencana site:.go.id |
|  |  | banjir OR badai OR bencana site:.org |
|  |  | water rescue site:.go.id |
|  |  | water rescue indonesia site:.org |
|  |  | (air OR perairan) penyelamatan site:.go.id |
|  |  | (air OR perairan) penyelamatan site:.gov |
|  |  | (kapal OR perkapalan OR layar OR pelayaran OR laut) kecelakaan site:.go.id |
|  |  | (kapal OR perkapalan OR layar OR pelayaran OR laut) kecelakaan site:.org |
|  |  | (kapal OR perkapalan OR layar OR pelayaran OR laut) keselamatan site:.go.id |
|  |  | (kapal OR perkapalan OR layar OR pelayaran OR laut) keselamatan site:.org |

## Appendix 2. Summary of studies reviewed

| **No** | **Authors** | **Year of study** | **Type of publication** | **Study aims** | **Data source** | **Study design** | **Sample, setting** | **Scale of study** | | **Types of unintentional drowning reported** | | | **Findings** | | | | | | |  | | |  | | |
| --- | --- | --- | --- | --- | --- | --- | --- | --- | --- | --- | --- | --- | --- | --- | --- | --- | --- | --- | --- | --- | --- | --- | --- | --- | --- |
|  |  |  |  |  |  |  |  | **National** | **Subnational** | **Accidental** | **Water-transport related** | **Disaster-related** | **Epidemiology** | **Risk factors** | | | | **Prevention** |  |  |  | | |  |  |
|  |  |  |  |  |  |  |  |  |  |  |  |  |  | **Sex** | **Age group** | **Location** | **Others** | **Intervention type** | **Relevant outcomes** |  | |  | | |  |
| 1 | Astreani and Alit (2015) | 2012-2014 | Original research article | To investigate cardinal signs of suspected drowning bodies | Medico-legal/ autopsy records | Observational descriptive | Drowning deaths recorded by the Forensic Department of Sanglah Provincial Hospital, Bali from 2012 to 2014 |  | √ | √ |  |  | A total of 112 drowning deaths was recorded by Sanglah Provincial Hospital of Bali between 2012-2014, with 91 cases included as samples on this study. | Male victims: 84.6% (n= 77/91), females: 15.4% (n=14/91). No measures of association were reported. | Adults: 87.9% (n=80/91); children: 12.1% (n=11/91). The definition of the term 'adult' and ' children' were not defined No measures of association were reported. | Beaches: 69.2% (n= 63/91), swimming pools: 13.2% (n=12/91), river: 13.2% (n=12/91), bathroom and swamps: 4.4% (n=4/91). No measures of association were reported. | Indonesians: 54.9% (n=50/91), foreign nationals: 45.1% (n=41/91). No measures of association were reported. | NA | NA |  | |  | | |  |
| 2 | Elsi and Gusti (2020) | 2020 | Original research article | To determine the fishermen's knowledge and attitude on Basic Life Support (BLS) for drowning victims. | Primary data | Cross sectional descriptive | Forty-one fishermen in Padang City, West Sumatra Province |  | √ | √ |  |  | NA | NA | NA | NA | Significant correlation between fishermen’s knowledge on BLS and attitude to BLS being given for drowning victims (p<0.05). | NA | NA |  | |  | | |  |
| 3 | Faradisi et al. (2021) | 2020 | Original research article | To provide education and training on first aid for cardiac arrest and respiratory arrest caused by drowning to street stallholders on coastal area | Primary data | Pre-test, post-test design | Twenty-one street stallholders on a coastal area of Nyamplung Beach, Pemalang Regency, Central Java Province |  | √ | √ |  |  | NA | NA | NA | NA | NA | Health education and roleplay on Cardio-Pulmonary Resuscitation (CPR) on drowning victims | Proportion of participants with good level of knowledge: post-intervention > pre-intervention. No measures of association were reported. |  | |  | | |  |
| 4 | Fernalia et al. (2022) | 2022 | Original research article | To form a water rescue-trained community group to assist during flooding, boating/shipping accident, or other drowning events | Primary data | Community service project | Community members at Lingkar Barat Subdistrict, Bengkulu Province (data on the number of participants are not available) |  | √ | √ | √ | √ | NA | NA | NA | Na | NA | Health education on performing water rescue. | Water rescue-trained local community group was formed. No evaluation on participants’ level of knowledge and skills on water rescue. No measures of association were reported. |  | |  | | |  |
| 5 | Gobel et al. (2014) | 2014 | Original research article | To determine the effect of health education on fishermen's level of knowledge on first aid for drowning victims | Primary data | Pre-test, post-test design | Forty-seven fishermen from North Bolaang Mongondow Regency, North Sulawesi Province |  | √ | √ |  |  | NA | NA | NA | NA | NA | Health education on first aid for drowning victims | Significant increase on the mean level of knowledge after the intervention applied (p<0.05).  No information on long-term knowledge retention. |  | |  | | |  |
| 6 | Hady et al. (2020) | Not stated | Original research article | To determine the effect of the roleplay method on the coastal area residents' level of knowledge and skills on performing first aid for drowning | Primary data | Pre-test, post-test design | Fifty people resided on a coastal area of Takalar Regency, South Sulawesi Province |  | √ | √ |  |  | NA | NA | NA | NA | NA | Health education and roleplay on first aid for drowning victims | There was a significance increase on the mean level of knowledge after the intervention applied (p<0.05) |  | |  | | |  |
| 7 | Hu et al. (2018) | 2016 | Original research article | To investigate frequency and intensity of floods, flood-induced mortality, and flood-affected population between 1975 and 2016 across the globe. | Secondary data | Analytical observational | Data on flood events worldwide were collected from: 1) the Emergency Disasters Database (EM-DAT), which contains information about each individual flood event during 1900-current; and 2) the Dartmouth Flood Observatory (DFO) covering a period of 1985-present | √ |  |  |  | √ | 1) Occurrence rate of floods, flood-induced mortality and flood-affected population were generally increasing globally; 2) The flood frequency and flood-induced mortality are the largest in Asia, specifically in China, India, Indonesia and the Philippines; 3) Flood-induced mortality rates for Indonesia between 1975 and 2016 were 1.0% to 3.0% of total flood-affected populations, with 5,000 to 10,000 flood-induced deaths reported for the study period. A possibility of selection and measurement/ information bias was identified. | NA | NA | NA | 1) The frequency of floods and flood-induced mortality were generally increasing globally, including in Indonesia; 2) Annual variation of mortality per flood event was highly related to floods with higher intensity, with the flood frequency and flood-induced mortality are the largest in Asia, including in Indonesia; 3) A large proportion of flood-induced deaths and the highest flood-induced mortality can be attributed to tropical cyclone-induced flash floods (no measures of association were reported); 4) Floodings and flood-induced deaths most often occurred in low-lying regions with dense river systems. Plain areas with slope of <0.5°: 40% of flood events and 50% of flood-induced deaths worldwide; plain areas with slopes 0.5°-15°: 60% of flood events and 48% of flood-induced deaths worldwide (no measures of association were reported); 5) Population density had a significantly positive correlation with the number of flood-related victims per unit area and number of deaths, while the flood-affected population and flood-induced mortality increased with the decrease of per capita GDP. | NA | NA |  | |  | | |  |
| 8 | Indonesian Ministry of Health (2020) | 2015-2019 | Grey literature (government report) | Accountability report: Directorate of Occupational and Sports Health, Indonesian Ministry of Health | NA | NA | NA | √ |  | √ | √ | √ | NA | NA | NA | NA | NA | A national level, coordinated policy development, regulatory activities, and social marketing framework on drowning prevention | The development of national drowning prevention framework is to be undertaken by the Directorate of Occupational and Sports Health (the Indonesian Ministry of Health), in coordination with the Indonesian Coordinating Ministry for Maritime and Investment Affairs; Ministry of Marine Affairs and Fisheries; Ministry of Youth and Sports Affairs; Ministry of Transportation; Ministry of Education and Culture; Ministry of Tourism; National Bureau of Statistics; Maritime Security Agency; Sea and Coast Guard; National Search and Rescue Agency; National Agency for Disaster Management; National Police, Seafarers Union; and Maritime Doctors Association.  Four main aspects of national drowning prevention framework to be initiated: a) Development of the National Drowning Prevention Coordinating Agency; b) Development of coordinated mechanisms of disseminating and monitoring health data; c) Development of the National Drowning Prevention Strategy; d) Development of drowning prevention awareness campaign targeting school-age children across Indonesia. |  | |  | | |  |
| 9 | Indonesian Ministry of Health (2017) | 2011 | Grey literature (policy statement of clinical standard) | National clinical practice guideline for treatment of trauma cases | NA | NA | NA | √ |  | √ | √ | √ | NA | NA | NA | NA | NA | Health information on pre-hospital and intrahospital emergency care for trauma patients | Mainly targeting Indonesian medical doctors |  | |  | | |  |
| 10 | Indonesian Ministry of Health (2015) | 2015 | Grey literature (policy statement) | Handbook on preventing drowning in children | NA | NA | NA | √ |  | √ |  |  | NA | NA | NA | NA | NA | Health information on risk factors and prevention of drowning in children | Targeting health workers and community members that had been through health education programme on giving first aid for drowning victims. |  | |  | | |  |
| 11 | Indonesian National Disaster Management Agency (2023) | 2014-2023 | Grey literature (government report) | Disaster information data | NA | NA | Data on frequency, number of deaths, and number of missing victims of flooding, flooding and avalanche and cyclone events covering a period of 1815 to 2023 | √ |  |  |  | √ | Between 1815 and 2023: 1) 13,927 flooding events with 22,476 deaths and 8,195 missing victims; 2) 9,503 flooding and avalanche events, with 3,324 deaths and 379 missing victims; 3) 499 tidal wave-related events, with 165 deaths and 49 missing victims; 4) 11,225 cyclone events, with 479 deaths and 49 missing victims. Excluding tsunami, these deaths contributed to 79.16% (N=100,434/126,875) of disaster-related deaths and 65.29% (N=8,672/13,283) of all disaster-related missing victims in Indonesia. No data on the cause of death. No information on data sources. A possibility of selection and measurement/ information bias was identified. | NA | NA | NA | NA | NA | NA |  | |  | | |  |
| 12 | Indonesian National Disaster Management Agency (2023) | 2013 - 2017 | Grey literature (government report) | Information on shipping/ boating accidents | NA | NA | Data on frequency, number of deaths, and number of missing victims of shipping/ boating accidents reported by the Indonesian Disaster Management Agency between February 2013 to April 2017 | √ |  |  | √ |  | Between 2014 to 2017, 15 shipping/boating accidents, with 121 deaths and 97 missing victims were reported. No information on the cause of deaths. A possibility of selection and measurement/ information bias was identified. | NA | NA | Open seas: 60% (n=9/15), rivers: 26.67% (n=4/15), lakes and during flooding events: 6.67% (n=1/15). No measures of association were reported. | Passenger boats: 86.67% (n/13/15), fishing boat: 6.67% (n=1/15), rescue boat: 6.67% (n=1/15. Boat overloading: 33.33% (n=5/15), collisions: 13.33% (n=2/15). No measures of association were reported. | NA | NA |  | |  | | |  |
| 13 | Lesmana et al. (2018) | 2018 | Original research article | To investigate coastal residents’ knowledge on first aid for drowning victims | Primary data | Pre-test, post-test design | Forty-six residents of a coastal area of Amal Beach, Tarakan City, North Kalimantan Province |  | √ | √ |  |  | NA | NA | NA | NA | NA | Health education on first aid on drowning victims | Proportion of participants with good level of knowledge: post-intervention > pre-intervention. No measures of association were reported. |  | |  | | |  |
| 14 | Nadapdap (2021) | 2021 | Grey literature (thesis) | To examine public knowledge of first aid for drowning victims | Secondary data | Literature review | Systematic search on 7 national journals and three international journals, using keywords "knowledge", "first aid", "drowning” |  | √ | √ |  |  | NA | NA | NA | NA | NA | Health education on BLS for drowning victims | Review identified respondents' knowledge as ‘good’ after being given health education on BLS for drowning victims. A possibility of selection bias and measurement bias was identified. No measures of association were reported. |  | |  | | |  |
| 15 | Nugroho and Suryono (2020) | 2019 | Original research article | To analyse the self-efficacy in emergency handling of toddler drowning victims | Primary data | Pre-test, post-test design | Fifteen members of a local freshwater fishing community of Darungan Village, Kediri, East Java Province |  | √ | √ |  |  | NA | NA | NA | NA | NA | Health education on first aid for infant drowning victims | Significant increase of the mean level of self-efficacy in emergency handling of toddler drowning victims after the intervention applied (p<0.05). No information on long-term knowledge and self-efficacy retention. |  | |  | | |  |
| 16 | Ose et al. (2020) | 2018 | Original research article | To determine the effect of health education on community health volunteers' level of knowledge on first aid for drowning victims and individuals with cardiac arrest | Primary data | Pre-test, post-test design | Thirty-two community health volunteers overseeing a coastal area of Amal Beach, Tarakan City, North Kalimantan Province |  | √ | √ |  |  | NA | NA | NA | NA | NA | Health education on performing CPR on drowning victims and individuals with cardiac arrest | Significant increase of the mean level of knowledge after the intervention applied (p<0.05). No information on long-term knowledge retention. |  | |  | | |  |
| 17 | Patimah (2019) | Not stated | Original research article | To determine the effect of health education on level of knowledge on BLS for drowning victims | Primary data | Pre-test, post-test design | Eighteen residents of a coastal area of Hamadi Village, Jayapura City, Papua Province |  | √ | √ |  |  | NA | NA | NA | NA | NA | Health education on BLS and evacuation on drowning victims | Significant increase of the mean level of knowledge after the intervention applied (p<0.05). No information on long-term knowledge retention. |  | |  | | |  |
| 18 | Patimah et al. (2019) | 2019 | Original research article | To determine community knowledge and attitudes on first aid for drowning victims | Primary data | Pre-test, post-test design | Fifty-eight residents of a coastal area of Hamadi Subdistrict, Jayapura City, Papua Province |  | √ | √ |  |  | NA | NA | NA | NA | NA | Health education on first aid on drowning victims | Proportion of participants with good level of knowledge: post-intervention > pre-intervention. No measures of association were reported. |  | |  | | |  |
| 19 | Pranoto et al. (2023) | 2022 | Original research article | To increase knowledge on assisting water accident victims | Primary data | Community service project | Sixty members of local tourism awareness group in Tua Pejat Village, Mentawai Islands, West Sumatra Province |  | √ | √ |  |  | NA | NA | NA | NA | NA | Health education on performing water rescue and CPR on accidental drowning victims | No evaluation on participants’ level of knowledge and skills of on water rescue and CPR. No measures of association were reported. |  | |  | | |  |
| 20 | Prasetyo (2017) | 2017 | Grey literature (thesis) | To investigate coastal area residents' level of knowledge on first aid for drowning victims | Primary data | Observational descriptive | Forty-seven residents living in a coastal area of Sulawesi Tenggara province |  | √ | √ |  |  | NA | NA | NA | NA | 'Sufficient' knowledge on first aid for drowning victims: 4.26% (n=2/47), 'insufficient' level of knowledge: 87.23% (n=41/47). No measures of association were reported. | NA | NA |  | |  | | |  |
| 21 | Rosmi et al. (2020) | 2017 | Original research article | To increase the knowledge on water rescue as an emergency response during flooding | Primary data | Community service project | Thirty-six members of youth organisations (Karang Taruna) in Benjeng Subdistrict, Gresik Regency, East Java Province |  | √ |  |  | √ | NA | NA | NA | NA | NA | Health education on the dangers of flooding and performing water rescue on flooding-related drowning victims | Water rescue-trained local community group was formed. No evaluation on participants’ level of knowledge and skills on flooding-related water rescue. No measures of association were reported. |  | |  | | |  |
| 22 | Sadewa et al. (2023) | 2022 | Original research article | To determine the feasibility of the utilisation of a modified a water rescue tool in water rescue efforts for drowning victims | Primary data | Descriptive quantitative and qualitative approach | Thirty-five participants consisting of lifeguards, pool attendants, swimming trainers, students, and swimming pool visitors at the Faculty of Sports and Health Sciences, Yogyakarta State University, Yogyakarta Province |  | √ | √ |  |  | NA | NA | NA | NA | NA | The development of modified water rescue tools (jerry cans lined with Styrofoam and equipped with ropes) for water rescue efforts, as an effective, cheap, easy to obtain substitute to water rescue equipment | On buoyancy, target accuracy, effectiveness and durability, the modified water rescue tool was deemed "very decent" by participants to be utilised on water rescue efforts. The definition of the term ‘very decent’ was not described. No evaluation on the implementation and outcome of the intervention. |  | |  | | |  |
| 23 | Saputra (2021) | 2003-2019 | Original research article | To analyse completed ship accident investigations | Secondary data | Descriptive observational | One hundred and twenty ship accidents investigations completed by the Indonesian National Transportation Safety Committee from 2003 until 2019. | √ |  |  | √ |  | Over the period of 2003 to 2019, completed investigations of a total of 120 ship accidents by the Indonesian National Transportation Safety Committee reported 513 deaths, 726 injured victims, and 701 missing victims. No information on the cause of deaths. | NA | NA | NA | Types of ship: 89% (n=107/120) occurred in motorboats (cargo ships, bulk carriers, container ships, and passenger ships (ferries and Ro-Ro ferries). Fire: 37% (n=44/120); submersion/sinking: 28% (n=34/120), collisions: 18% (n=22/120), other causes: 17% (n=20/120). Contributing factors to shipping accidents: 1) poor maintenance of ships; 2) unavailability/ poor maintenance of safety equipment on board; 3) poor knowledge, awareness, and compliance of safety regulations; and 4) underqualified seafarers and poor ship crews' capacity in ensuring safe shipping/maritime practice. No measures of association were reported. | NA | NA |  | |  | | |  |
| 24 | Sillehu and Kartika (2018) | 2015 | Original research article | To investigate the role of the Indonesian National Search and Rescue Agency in rescuing drowning victims in sea | Primary data | Analytical observational | Thirty-five regency-level National Search and Rescue Agency staff members for Ambon City, Maluku Province |  | √ | √ | √ |  | NA | NA | NA | NA | NA | No intervention | Significant correlation between the performance by the National Search and Rescue Agency and the rescue of drowning victims (p<0.05). A possibility of selection bias and measurement bias was identified |  | |  | | |  |
| 25 | Sugiantoro and Wahyudi (2021) | 2020 | Original research article | To examine the effect of health information on knowledge and attitude on first aid for drowning | Primary data | Pre-test, post-test design | Fifteen fishermen from Lempasing Regency of Lampung Province |  | √ | √ | √ |  | NA | NA | NA | NA | NA | Health information on first aid for drowning victims | Significant increase of the mean level of knowledge and attitude after the intervention applied (p<0.05). No information on long-term knowledge retention. |  | |  | | |  |
| 26 | Sukarna et al. (2021) | 2020 | Original research article | To determine the effect of health education on knowledge and skills on evacuating drowning victims | Primary data | Pre-test, post-test design | Thirty street stallholders on a coastal area of Sijuk, Belitung Regency, Bangka Belitung Islands Province |  | √ | √ |  |  | NA | NA | NA | NA | NA | Health education on performing CPR and evacuation on drowning victims | Significant increase of the mean level of knowledge after the intervention applied (p<0.05). No information on long-term knowledge retention. |  | |  | | |  |
| 27 | Suryono and Nugroho (2020) | Not stated | Original research article | To determine the effect of health education on knowledge and skills on performing first aid on infant drowning victims | Primary data | Pre-test, post-test design | Fifteen members of a local freshwater fishing community of Darungan Village, Kediri Regency, East Java Province |  | √ | √ |  |  | NA | NA | NA | NA | NA | Health education on first aid for infant drowning victims | Proportion of participants with sufficient level of knowledge: post-intervention > pre-intervention. No measures of association were reported. |  | |  | | |  |
| 28 | Suwardjo et al. (2010) | 2006-2008 | Original research article | To assess fatality accident rate (FAR) of fishing vessel accidents around Tegalsari, Pekalongan and Cilacap Fishing Ports of Central Java Province | Primary and secondary data | Analytical observational | Data on 61 fishing vessel accidents collected from the harbourmasters of Tegalsari, Pekalongan and Cilacap Fishing Ports; the Indonesian Fishermen Association; the Indonesian Water Police Unit; and the Indonesian Department of Maritime Affairs and Fisheries between 2006 and 2008 |  | √ |  | √ |  | Sixty-one fishing vessel accidents were recorded to occur around the three fishing ports in Central Java Province between 2006 and 2008. A total of 68 deaths, or an annual average of 32 dead/missing fishing crews, was reported: 26.48% (n=18/68) men overboard during shipping/fishing, 45.59% (n=31/68) due to ship capsizing (cause of deaths was not stated). A total of 22, or an annual average of 7, drowned/missing fishing vessels was reported. The average FAR was 115 deaths/100,000 fishermen. |  |  |  | Contributing factors to fishing vessel accidents: 1) underqualified shipping crews; 2) poor knowledge, awareness, and compliance of safety regulations; 3) unfulfillment of safety requirements. Approximately 84.3% of skippers and ship crews did not go beyond primary level education, hence did not qualify to undertake Basic Safety Training for shipping crews. Seventy per cent (n=45/64) of all fishing vessels registered did not fulfil safety requirements due to insufficient number of life jackets and rescue buoys, unequipped with fire extinguishers and life rafts, and lacking in other safety equipment.  The highest numbers of fishing vessel accidents were recorded during rainy seasons (November to February). No measures of association were reported. | NA | NA |  | |  | | |  |
| 29 | Usaputro and Yulianti (2013) | 2010-2012 | Original research article | To determine the characteristics and risk factors of drowning deaths | Medico-legal/ autopsy records | Observational descriptive | Drowning deaths recorded by the Forensic Department of Sanglah Provincial Hospital of Bali between 2010 and 2012 |  | √ | √ |  |  | A total of 97 drowning deaths was recorded by Sanglah Provincial Hospital of Bali between 2010-2012, with 71 cases included as samples in this study. | Male victims: 84,5% (n= 60/71), females: 15,5% (n=11/71). No measures of association were reported. | Aged 21–30 years: 22.5% (n=16/71), >50 years: 19.7% (n=14/71), 31–40 years: 18.3% (n=13/71), <20 years: 16.9% (n=12/71). No measures of association were reported. | Open seawater: 53.5% (n= 38/71), freshwater bodies: 25.4% (n=18/71), unknown location: 21.1% (n=15/71). No measures of association were reported. | Foreign nationals: 49.3% (n=35/71), Indonesians: 40.8% (n=29/71). Blood alcohol was identified in 20%(n=4/20) (no information on the blood alcohol content), fatal trauma in 20% (n=4/20), and history of comorbid condition(s) in 15% (n=3/20) (no information on comorbid conditions) of autopsied drowning victims. No measures of association were reported. | NA | NA |  | |  | | |  |
| 30 | Welembuntu et al. (2021) | 2021 | Original research article | To investigate the residents' knowledge on first aid for maritime accidents' victims | Primary data | Observational descriptive | Forty residents of coastal Kulur II Village, Tabukan Tengah Subdistrict, Sangihe Islands Regency, North Sulawesi Province |  | √ |  | √ |  | NA | NA | NA | NA | ‘Sufficient’ level of knowledge on first aid for maritime accident victims: 55% (n=22/40), ‘good’ level of knowledge: 42.5% (n=17/40). No measures of association were reported. | NA | NA |  | |  | | |  |
| 31 | Widyastuti and Rustini (2017) | 2017 | Grey literature (conference proceeding) | To investigate the coastal area community knowledge on first aid for drowning victims | Primary data | Observational descriptive | Thirty-five residents of a coastal area of Bulak Subdistrict, Surabaya City, East Java Province |  | √ | √ |  |  | NA | NA | NA | NA | ‘Sufficient’ level of knowledge on first aid for drowning victims: 57.14% (n=20/35), 'good' level of knowledge: 31.4% (n=11/35), 'poor' level of knowledge: 11.4% (n=4/35). No measures of association were reported | NA | NA |  | |  | | |  |
| 32 | Wulur (2013) | 2007-2011 | Original research article | To examine autopsy findings in drowning cases at Prof. Dr. R. D. Kandou Provincial Hospital of North Sulawesi between January 2007 and December 2011 | Primary data | Observational descriptive | Drowning deaths recorded by the Forensic Department of Prof. Dr. R. D. Kandou Provincial Hospital of North Sulawesi, between January 2007 and December 2011 |  | √ | √ |  |  | A total of 15 drowning deaths was recorded by Prof. Dr. R. D. Kandou Provincial Hospital of North Sulawesi between 2007-2011. All cases identified were included as samples for this study. | Male victims: 80% (n= 12/15), females: 20% (n=3/15). No measures of association were reported. | Adults aged ≥ 20 years: 86.7% (n=13/15), children aged 5-14 years: 6.67% (n=1/15). No measures of association were reported. | NA | NA | NA | NA |  | |  | | |  |
